# Insight in modulation of inflammation in response to diclofenac intervention: a human intervention study

**DOI:** 10.1186/1755-8794-3-5

**Published:** 2010-02-23

**Authors:** Marjan J van Erk, Suzan Wopereis, Carina Rubingh, Trinette van Vliet, Elwin Verheij, Nicole HP Cnubben, Theresa L Pedersen, John W Newman, Age K Smilde, Jan van der Greef, Henk FJ Hendriks, Ben van Ommen

**Affiliations:** 1 TNO Quality of Life, PO Box 360, 3700 AJ Zeist, the Netherlands; 2 Current address: CCMO, The Hague, the Netherlands; 3 USDA, ARS, Western Human Nutrition Research Center, 430 West Health Sciences Dr, Davis, CA 95616, California, USA; 4 Department of Nutrition, University of California, Davis, California, USA; 5 Current address: Biosystems Data Analysis, Swammerdam Institute for Life Sciences, University of Amsterdam, Nieuwe Achtergracht 166, 1018 WV Amsterdam, the Netherlands

## Abstract

**Background:**

Chronic systemic low-grade inflammation in obese subjects is associated with health complications including cardiovascular diseases, insulin resistance and diabetes. Reducing inflammatory responses may reduce these risks. However, available markers of inflammatory status inadequately describe the complexity of metabolic responses to mild anti-inflammatory therapy.

**Methods:**

To address this limitation, we used an integrative omics approach to characterize modulation of inflammation in overweight men during an intervention with the non-steroidal anti-inflammatory drug diclofenac. Measured parameters included 80 plasma proteins, >300 plasma metabolites (lipids, free fatty acids, oxylipids and polar compounds) and an array of peripheral blood mononuclear cells (PBMC) gene expression products. These measures were submitted to multivariate and correlation analysis and were used for construction of biological response networks.

**Results:**

A panel of genes, proteins and metabolites, including PGE_2 _and TNF-alpha, were identified that describe a diclofenac-response network (68 genes in PBMC, 1 plasma protein and 4 plasma metabolites). Novel candidate markers of inflammatory modulation included PBMC expression of annexin A1 and caspase 8, and the arachidonic acid metabolite 5,6-DHET.

**Conclusion:**

In this study the integrated analysis of a wide range of parameters allowed the development of a network of markers responding to inflammatory modulation, thereby providing insight into the complex process of inflammation and ways to assess changes in inflammatory status associated with obesity.

**Trial registration:**

The study is registered as NCT00221052 in clinicaltrials.gov database.

## Background

In obesity, both the adipose tissue mass and the extent of macrophage infiltration increase. Moreover, both adipocytes and macrophages can release a range of inflammatory markers, thereby contributing to a local and systemic state of "low-grade" inflammation [1,2]. The systemic inflammatory status often seen in obese subjects is associated with development of obesity-related diseases like cardiovascular diseases [3,4], diabetes mellitus and insulin resistance [5-7]. Modulation of inflammation in overweight subjects may be a means to reduce the risk of diseases associated with obesity.

However, it is difficult to detect modulation of inflammation, as inflammation is a complex process that can be poorly described with a single marker. In fact, a range of markers for local and systemic inflammation have been described and evaluated, primarily with respect to the risk of atherosclerosis and cardiovascular disease development. As with classic inflammatory conditions, obesity is associated with elevated levels of the acute phase reactant C-reactive protein (CRP) [8]. CRP is a well described marker of risk for development of both coronary heart disease [9] and type-2 diabetes [10]. Other inflammatory markers include cytokines such as interleukin-6 (IL-6), which can regulate CRP release [11,12], and tumor necrosis factor alpha (TNF-alpha), adhesion molecules such as VCAM-1, ICAM-1 and E-selectin [13] and eicosanoids like prostaglandin E2 (PGE_2_) [14,15]. In type-2 diabetes, levels of multiple inflammatory markers (CRP, IL-6, adhesion molecules) are elevated in an early disease stage and increased further with disease progression [16]. Plasma sialic acid level also rises in association with metabolic syndrome, including insulin resistance and type-2 diabetes, and appears to be a marker of acute micro vesicular endothelial damage [17-19].

The focus of the present study was to investigate and provide insight into the modulation of obesity-associated inflammation, by applying a mild anti-inflammatory therapy to overweight males. Diclofenac, a non-steroidal anti-inflammatory drug (NSAID), which is known to inhibit prostaglandin synthesis by inhibition of cyclo-oxygenase enzymes, was chosen as a model compound. Due to the complexity of the inflammatory process, we used a wide-range of 'omics-based' parameters to extensively characterize modulation of inflammation. This analysis approach included measurements of 80 plasma proteins, more than 300 plasma metabolite levels (lipids, free fatty acids, oxylipids and a wide array of polar compounds) and whole genome expression profiles in PBMCs.

Analysis of the 'omics-based' datasets was performed using multivariate and correlation analysis. The results were used to construct biological networks, providing visualization of the interactions between identified markers. This integrated inspection provided new insights into the complex process of inflammation and ways to assess changes in inflammatory status associated with obesity.

## Methods

### Subjects and study design

The study was conducted at TNO Quality of Life (Zeist, the Netherlands). Overweight or mildly obese men with a body mass index (BMI) between 25.1 and 34.0 kg/m^2 ^were recruited from a pool of volunteers. Fifty subjects gave written informed consent after being informed about the study, both verbally and in writing. All subjects completed a questionnaire on medical history and were submitted to a physical examination. Blood and urine were collected after an overnight fast for routine analysis. In addition, plasma CRP levels were determined using a high-sensitivity CRP assay.

Subjects who, based on medical histories, were not suitable to receive diclofenac treatment (history of current gastro-intestinal diseases including bleeding, ulcer or perforation, history of stroke, history of current significant hematological disorders, any significant hepatic, renal or cardiovascular disease or asthma) and subjects with allergy or hypersensitivity for non-steroidal anti-inflammatory drugs (NSAIDs) were excluded from participation. Furthermore, smokers and subjects who reported slimming or who were on a medically prescribed diet were excluded from participation. Also, subjects who were on medication that may have interfered with parameters to be measured or with diclofenac treatment and subjects with a history of medical or surgical events that may have affected the study outcomes were not included. Based on these criteria, twenty five subjects were eligible. Twenty overweight men (25 < BMI <31) were selected on the basis of the highest CRP values and nineteen completed the study. One person dropped out on the first day of the study for study unrelated reasons. Subject characteristics are presented in table 1.

**Table 1:** Demographic data of subjects that completed the study (n = 19) at screening.

	All (n = 19)	Placebo treatment (n = 10)	Diclofenac treatment (n = 9)
Age (years)	43 ± 15	41 ± 16 (19 - 60)	45 ± 15 (21 - 58)
Body weight (kg)	93.5 ± 8.0	93.5 ± 9.3 (81.1 - 105.2)	93.5 ± 6.9 (85.2 - 104.4)
Height (m)	1.82 ± 0.08	1.82 ± 0.10 (1.69 - 1.96)	1.83 ± 0.07 (1.70 - 1.92)
BMI (kg/m^2^)	28.1 ± 1.2	28.1 ± 1.0 (26.7 - 29.3)	28.1 ± 1.5 (26.1 - 30.9)
hs-CRP (mg/L)	2.22 ± 2.33	2.08 ± 1.88 (0.41 - 6.35)	2.37 ± 2.87 (0.64 - 9.72)
Fasting glucose (mmol/L)	6.0 ± 0.5	5.9 ± 0.5 (5.2 - 7.1)	6.0 ± 0.6 (5.0 - 6.8)
Fasting insulin (mU/L)	13.4 ± 8.1	13.4 ± 8.6 (5.1 - 26.8)	13.3 ± 8.1 (3.3 - 26.6)

Values: mean ± SD (range)

The study was approved by the Medical Ethics Committee of the University Medical Centre of Utrecht (May, 2005) and conducted according to the current assembly (52nd) of the Declaration of Helsinki (Edinburgh, Scotland, October 2000) and the ICH Guideline for Good Clinical Practice (ICH Topic E6, adopted 01-05-1996 and implemented 17-01-1997). The study is registered as NCT00221052 in clinicaltrials.gov database.

The study was designed as a double blind, randomized, parallel trial, in which subjects were treated with diclofenac (n = 9) or placebo (n = 10). Randomization of subjects to treatment groups was restricted by CRP, BMI, fasting glucose and age. Subjects consumed one capsule (placebo or 50 mg diclofenac) approximately one hour before breakfast, lunch and dinner for 9 days. A treatment period of 9 days was chosen as suitable treatment duration to both detect anti-inflammatory effect and avoid side effects as much as possible. The dose of 150 mg/day is a common dose prescribed in osteoarthritis or rheumatoid arthritis (e.g [20]).

Subjects were instructed to keep their habitual diet during the study. Blood samples were taken after an overnight fast on day 0, 2, 4, 7, 9 and 10. Subjects underwent an oral glucose tolerance test (OGTT) on day 0 and day 9 (data discussed in [21]).

### Measurement of PGE_2_, CRP and sialic acid levels

Levels of PGE_2_, hsCRP and sialic acid were measured in fasting plasma. PGE_2 _was determined using the Prostaglandin E_2_(^125^I) Biotrak assay system (Amersham Biosciences, UK) with modifications. In short, PGE_2 _in samples was derivatized to the methyl oximate derivative. The resulting solution was further diluted (final dilution 5 times) in PBS and assayed. The assay consists of incubation of the oximated sample PGE_2_, the ^125^I-labelled PGE_2_, and a PGE_2 _specific antibody. After incubation, the Amerlex-M reagent is added and the free and bound ^125^I labeled PGE_2 _separated using centrifugation. The resulting bound radioactivity in the pellet is determined using a gamma-counter. One subject (number 7) was excluded from the PGE_2 _analysis since day 0 value was missing due to an analytical problem.

CRP levels were determined using the CRP ELISA kit 'CRP sensitiv' (Immun Diagnostik, Bensheim, Germany) according to the manufacturer's instructions. The enzymatic determination of sialic acid (N-acetyl-neuraminic acid) was performed using the colorimetric assay by Roche Diagnostics (Mannheim, Germany).

Data were analyzed for time and treatment effects using 2-way ANOVA in SAS v9 (SAS Institute Inc., Cary, USA).

### RNA isolation, labeling and hybridization

Peripheral blood mononuclear cells (PBMCs) were isolated from fasting blood samples taken on day 0 and day 10. Blood was transferred to Leucosep tubes filled with Fycoll and centrifuged at 800 × g for 20 min. Then, the PBMC layer was transferred to a clean tube and after two steps of washing with PBS and centrifugation at 225 × g for 10 min, PBMCs were resuspended and stored at -70°C. RNA was isolated from PBMCs using NucleoSpin columns (Bioké, Leiden, the Netherlands) according to the manufacturer's instructions. Integrity of RNA obtained was examined by Agilent Lab-on-a-chip technology using the RNA 6000 Nano LabChip kit and a bioanalyzer 2100 (Agilent Technologies, Amstelveen, the Netherlands).

The isolated RNA samples were sent to ServiceXS BV (Leiden, the Netherlands) where they were processed according to Affymetrix protocols. In brief, RNA concentration was determined by absorbency at 260 nm, and quality and integrity was verified using the RNA 6000 Nano assay on the Agilent 2100 Bioanalyzer (Agilent Technologies).

Next, 2 μg of high quality total RNA was used with the Affymetrix Eukaryotic One-Cycle Target Labeling and Control reagents to generate biotin-labeled antisense cRNA. The quality of the cRNA was checked using the Agilent 2100 bioanalyzer. The labeled cRNA was further used for the hybridization to Affymetrix Human Expression U133 2.0 Genechips (with 54613 probesets). After an automated process of washing and staining, absolute values of expression were calculated from the scanned array using the Affymetrix GCOS software.

### Transcriptome data analysis

Quality control and normalization of microarray data was performed using R/BioConductor packages through the NuGO MadMax pipeline https://madmax.bioinformatics.nl, which is also available as a Genepattern procedure on http://nbx2.nugo.org[22]. One array did not pass the quality control, due to high background values and the data for this subject from the diclofenac group was excluded from the transcriptome data analysis.

Raw signal intensities from CEL files were normalized using the GCRMA algorithm. Normalized signal intensities below 10 were replaced by 10 and only probesets with at least one signal intensity value >15 were included in further data analysis (24833 probesets). Data were log (base2) transformed and a 2-way ANOVA was performed using SAS v9 (SAS Institute Inc., Cary, USA) to assess the time × treatment interaction effects. To reduce number of genes in the multivariate analysis and thus the skewness of the dataset, genes with *p *< 0.1 for time × treatment interaction in the ANOVA (3355 genes) were selected and analyzed further using PLS-DA using Matlab Version 7.0.4 R14 (The Mathworks, Inc.) as described below under *Multivariate analysis of 'omics' data*.

Subsets of genes selected by PLS-DA were analyzed in GenMAPP v2.0 http://www.genmapp.org to find functional groups of genes (based on Gene Ontology) that were overrepresented in these subsets [23]. Transcriptome data are available through ArrayExpress (E-TABM-740).

### Proteomics analysis

Blood collected in the presence of EDTA provided plasma samples, which were shipped to Rules Based Medicine, Inc. (Austin, USA). Measurement of expression levels of 79 proteins (HumanMAP, antigen panel) was conducted using fully automated, bead-based multiplex sandwich immunofluorescence assays (see list of proteins in additional file 1).

Some of the variables in the proteomic data set contained a high number of measurements below the detection limit which could seriously disturb the statistical analysis. Therefore, the so-called 80% rule [24] was applied to retain only those peaks which have 80% or more values above the detection limit for at least one of the two treatment groups, resulting in retention of 64 out of the 79 variables. Although they did not fulfill the criteria of the 80%-rule, TNF-alpha (65% and 67% of values above detection limit in placebo group and diclofenac group, respectively) and matrix metallopeptidase 9 (MMP-9) (65% and 78% of values above detection limit in placebo group and diclofenac group, respectively) were also retained due to their known role in inflammation, resulting in 66 proteomic variables being included for statistical analysis. Values below the detection limit that remained in the truncated data set were replaced by a value of half of the lower assay limit.

### Metabolomics analysis

The analysis of plasma samples for lipids, free fatty acids (FFA) and "polar" metabolites by liquid chromatography-mass spectroscopy (LC-MS) and "global" metabolite assessments by GC-MS and prepocessing of the metabolic profiling data are described in detail by Wopereis et al. [21]. The LC-MS FFA data set contained 14 annotated peaks, the LC-MS Lipids data set existed of 61 annotated peaks, 120 metabolites were included in the LC-MS polar data set and the GC-MS global data set contained 137 metabolites.

### Oxylipids analysis

The oxygenation of polyunsaturated fatty acids yields a wide variety of compounds with potent inflammatory and anti-inflammatory properties (e.g. prostaglandins). In this study, plasma oxylipids were quantified using modification of published procedures [25]. Specifically, samples were randomized and extracted using 60 mg Oasis HLB (Waters Corporation, Milford, MA) solid phase extraction (SPE) cartridges along with a laboratory plasma reference material, a supplied replicate sample and a blank of pH 7.4, 0.1 M phosphate buffered saline solution (PBS). SPE cartridges were pre-washed then conditioned with 3.5 mL with 0.1% acetic acid in 5% methanolic water. Samples and controls (250 μL) were placed in independent SPE solvent reservoirs, then spiked with deuterated analytical surrogates and an anti-oxidant/chelator mix (EDTA and butylated hydroxy toluene), then allowed to equilibrate for 2 min. Samples were diluted with 1 mL of 0.1% acetic acid:5% MeOH solution, drawn through SPE cartridges by light vacuum, washed with 3.5 mL 0.1% acetic acid:5% MeOH solution and sorbent was dried under 20 psi vacuum. Analytes of interest were eluted with MeOH followed by ethyl acetate into polypropylene tubes containing 2 μL glycerol. Solvents were removed under vacuum and residues stored at -20°C until day of analysis. Upon analysis residues were reconstituted with 50 μL of 800 nM 1-cyclohexyl-ureido-3-dodecanoic acid (CUDA) in MeOH and filtered with Durapore^® ^PVDF 0.1 mm spin-filter tubes (Millipore, Billerica, MA, USA), at 4°C, then transferred to autosampler vials.

Analytes from sample extracts (10 μL) were separated by reverse phase gradient (A: H20 w/0.1% acetic acid and B: 90:10 acetonitrile/isopropanol (v/v) at 250 μL/min) on a UPLC using a 2.1 × 150 mm HSS-C18 Acquity column (Waters Corp.). Oxylipids and fatty acids were detected by negative mode electrospray ionization on a Quattro Micro (Waters Corp.) tandem mass spectrometer. Analytes were quantified with QuanLynx v.4.0 software (Waters) using internal standard methodologies against a minimum 5 pt calibration curve bracketing all reported concentrations.

### Multivariate analysis of 'omics' data

Partial Least Squares Discriminant analysis (PLS-DA; [26]) was used to identify genes, proteins and metabolites that differed in their change between day 0 and day 9 between the diclofenac and placebo group. Data were mean-centered for analysis. In PLS-DA, a Y-variable containing class membership information is correlated to a data matrix (X-block). The subjects who received the placebo and diclofenac treatments were assigned to class '0' and class '1', respectively. The X-block was defined for each data set (genes, proteins and metabolites per platform) by subtracting the day 0 values from the day 9 values, and a PLS-DA model was made for each data set. The largest number of PLS factors that was considered was 10.

The PLS-DA models were evaluated using a 'leave-one-out' cross-validation scheme [27], such that data from one subject was left out in the first cross-validation step, a PLS-DA model was built, and the treatment class membership of removed subject was predicted. The process was repeated until all 19 subjects were left out once. The error rate of the model was determined by comparing the original class membership and the predicted one. The optimal number of latent variables (LVs) was determined based on the minimum value of this error rate and the final fit of the model was made using this number of optimal LVs.

PLS-DA models with error rates below 30% were optimized by performing variable selection based on a jackknife approach. In this procedure, data of one subject was left out and a PLS-DA model was made using the same number of LVs that was used for the final model. This was repeated until all 19 subjects were left out once, resulting in 19 sets of regression coefficients, the standard deviation of which was used to determine the relative standard deviations (RSDs) of each regression coefficient. Only those variables with RSDs <50% were included in a truncated data set used to build a second PLS-DA model. Components that contributed to treatment differences were identified based on the absolute regression coefficients of this model. All multivariate data analyses were performed using Matlab Version 7.0.4 R14 (The Mathworks, Inc.).

### (Combined) data analysis & interpretation

Differences between response to diclofenac and placebo treatment was analyzed by PLS-DA (as described above) and 2-way ANOVA. Markers which increased or decreased at least 20% in 6 or more subjects in the diclofenac group were considered specifically responsive to diclofenac intervention. Markers that showed a >20% change in the same direction in 6 or more subjects in the placebo group were disregarded. The changes in selected markers are displayed as a heatmap.

Correlation analysis was performed in SAS Enterprise Guide v. 4.1 (SAS Institute Inc., Cary, USA). The significance threshold was set to *p *< 0.01. Biological networks were generated using curated interactions in MetaCore v4.7 (GeneGo Inc., St. Joseph, MI, USA). MetaCore™ is based on a proprietary manually curated database of human protein-protein, protein-DNA and protein compound interactions, metabolic and signaling pathways and the effects of bioactive molecules in gene expression. Lists of genes, proteins and metabolites that responded to diclofenac and lists of genes, proteins and metabolites with highest correlation to CRP changes were uploaded into MetaCore™ for construction of biological networks based on known interactions. Pathway maps were edited in Mapeditor (GeneGo Inc., St. Joseph, MI, USA) version 2.6.0.

## Results

### Inflammatory markers

In the current study modulation of inflammation by diclofenac was investigated by integrated analysis of the response of genes in peripheral blood mononuclear cells (PBMC) and of proteins and metabolites in plasma, including known inflammatory markers, in order to investigate modulation of inflammatory status in state of obesity.

Firstly, effects of diclofenac on known inflammatory markers CRP, PGE_2 _and sialic acid were assessed. While sialic acid levels were not different between groups, responses of PGE_2 _and CRP on diclofenac treatment differed significantly from responses on placebo treatment (PGE_2_: treatment × time interaction *p *= 0.017; hsCRP: treatment × time interaction *p *= 0.0273).

**Figure 1:**
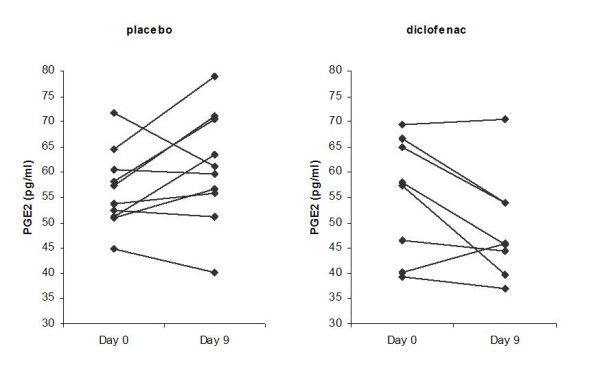
**PGE2 levels before (day0) and after (day9) supplementation with placebo and diclofenac**. The PGE2 response was significantly different for diclofenac compared to placebo (ANOVA, treatment * time interaction P < 0.05). In the diclofenac group, the difference in PGE2 level between day 9 and day 0 was significant (p-value = 0.0469).

**Figure 2:**
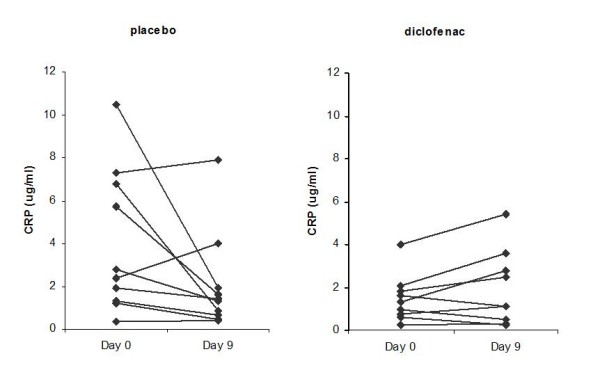
**CRP levels before (day0) and after (day9) supplementation with placebo and diclofenac**. The CRP response was significantly different for diclofenac compared to placebo (ANOVA, treatment * time interaction P < 0.05). In the placebo group, hsCRP levels were significantly different at day 9 compared to day 0 (P = 0.0062).

As expected, diclofenac treatment decreased PGE_2 _levels (48.9 ± 10.6 pg/mL (mean ± stdev) at day 9 compared to 55.3 ± 11.9 pg/mL at day0, *p *= 0.047), as displayed in figure 1. In contrast, CRP levels were significantly reduced in the placebo group (2.05 ± 2.30 μg/mL (mean ± stdev) at day 9 compared to 4.03 ± 3.34 μg/mL at day0, *p *= 0.0062), but not in the diclofenac group. As illustrated in figure 2, four subjects in the placebo group exhibited elevated CRP levels (>5 μg/mL) at day 0. The CRP levels in three of these subjects dropped below 2 μg/mL at day 9, resulting in a 3.6 to 8 fold reduction in CRP levels at day 9 compared to day 0.

### Combined analysis of diclofenac treatment effects

In addition to assessment of classical markers, changes in PBMC transcriptome, plasma proteome and plasma metabolome were assessed. Transcriptomic, proteomic and metabolomic datasets were submitted to supervised multivariate analysis (PLS-DA) to identify differences in response to diclofenac treatment compared to placebo. The results of the analysis are shown in table 2. With respect to metabolomic datasets, a significant difference between diclofenac and placebo treatment was detected only for metabolites from the oxylipid metabolomics platform: a set of 19 oxylipids were retained in a model with 10.5% error rate (see additional file 2). More detailed analysis of metabolomics data showed that, for other metabolomics platforms (GC-MS global, LC-MS polar, LC-MS lipids and LC-MS free fatty acids) changes were only detected in combination with an oral glucose tolerance test [21]. In addition to oxylipids, plasma protein and PBMC gene expression data also indicated differential signatures between diclofenac and placebo groups. The selection of 46 plasma proteins is listed in additional file 3. The selection of 3355 genes was submitted to enrichment analysis, to assess which biological processes are involved in the differential response between diclofenac and placebo intervention. Almost all top-ranked processes were inflammation-related, such as defense response, immune response, inflammatory response and response to stress (see additional file 4 for a complete list of enriched biological processes).

**Table 2:** Overview of results of multivariate analysis (PLS-DA) of various data sets.

	Original data set		After variable selection	
	# variables	error rate	# variables	error rate
plasma proteomics	66	21%	46	10%
plasma oxylipids	30	26%	19	10.5%
plasma GC-MS	137	42%	-	-
plasma LC-MS polar	130	53%		
plasma LC-MS lipids	61	37%	-	-
plasma LC-MS free fatty acids	14	37%	-	-
PBMC transcriptomics	-	-	3355	0%

All data sets were analyzed for differences between diclofenac and placebo treatment by PLS-DA. The results of the different multivariate models are expressed as error rates. Only when the error rate for the original data set was < = 30%, analysis was continued by variable selection, as described in the materials and methods section.

**Figure 3:**
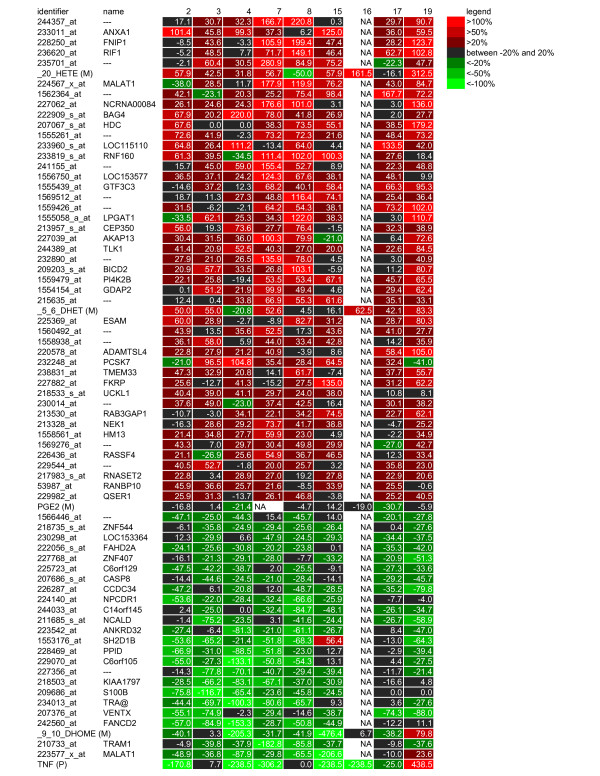
**Heatmap of responses of 68 genes, 1 protein (P) and 4 metabolites (M) to diclofenac intervention**. Numbers represent % change in each subject in response to diclofenac. Each row lists identifier and name. For genes identifiers are Affymetrix probeset IDs, for proteins and metabolites names are shown followed by (P) or (M), respectively.

**Figure 4:**
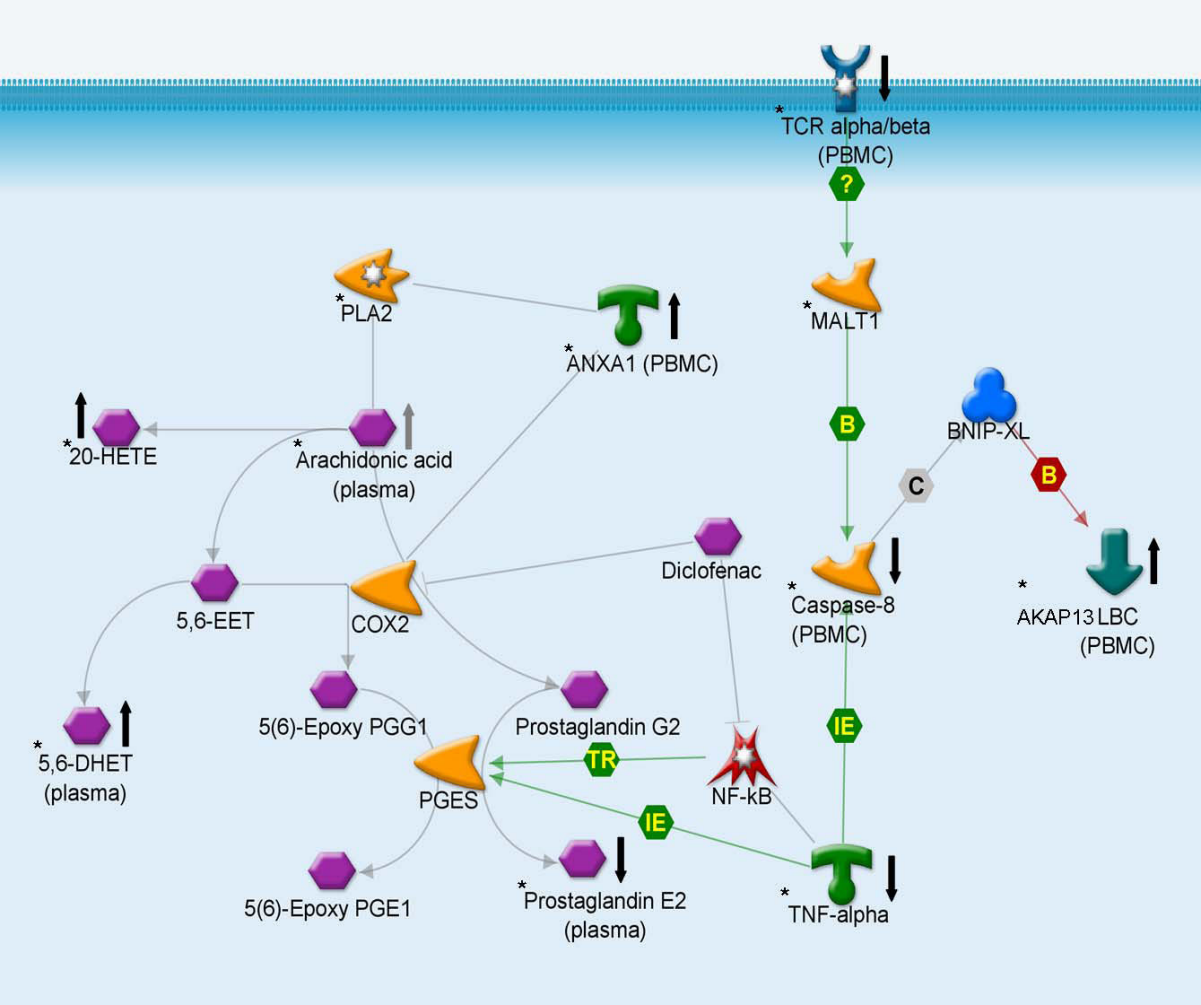
**Network of response related to diclofenac intervention, generated using MapEditor from GeneGo Inc**. * indicates markers measured in the current study. Arrow pointing upward indicates increase in response to diclofenac treatment, arrow pointing downward indicates decrease in response to diclofenac treatment. PLA2: phospholipase A2; ANXA1: annexin A1; COX2: cyclooxygenase 2; 20-HETE: 20-hydroxyeicosatetraenoic acid; 5,6-EET: 5(6)-epoxyeicosatrienoic acid; 5,6-DHET: 5,6-dihydroxy-eicosatrienoic acid; PGG1: prostaglandin G1; PGE1: prostaglandin E1; PGES: prostaglandin E synthase; TCR: T cell receptor; MALT1: mucosa-associated lymphoid tissue lymphoma translocation protein 1; BNIP-XL: Protein prune homolog 2; LBC: A-kinase anchor protein 13 (AKAP13). IE: influence on expression, B: binding.

A primary goal of this study was to identify markers for modulation of inflammation in reponse to diclofenac intervention. The inflammatory modulation by diclofenac was investigated by detailed analysis of responses of genes, proteins and oxylipids selected by multivariate analysis. Markers that respond specifically to diclofenac intervention were selected based on >20% increase or >20% decrease in at least 6 subjects treated with diclofenac (as described above in *(Combined) data analysis & interpretation*). To focus on modulation of inflammation by diclofenac, and not acute phase response, only markers that did not show significant correlation to CRP response (across all subjects) were selected. This resulted in the selection of 68 genes, 1 protein and 3 oxylipids. The expression changes of these markers are visualized in a heatmap (Figure 3), together with changes in PGE_2_. In addition to changes in PGE_2_, diclofenac intervention also altered levels of other plasma oxylipids, increasing 5,6-dihydroxy-eicosatrienoic acid (5,6-DHET) and 20-HETE (20-hydroxyeicosatetraenoic acid) and decreasing the linoleate derived 9,10-dihydroxyoctadecenoic acid (9,10-DHOME). The arachidonic acid metabolite 5,6-DHET is a stable hydrolysis product of the 5(6)-epoxyeicosatrienoic acid (5(6)-EET) (not measured in current study) which itself is a substrate of cyclooxygenase leading to formation of epoxy prostaglandins. Levels of arachidonic acid (measured in FFA metabolomics analysis) were increased in the diclofenac group at day 9 compared to day 0, but the responses in diclofenac and placebo treatment groups were not significantly different (data not shown). Diclofenac intervention resulted in decreased levels of TNF-alpha. Furthermore, the selection of genes includes genes with a known role in inflammation like T cell receptor alpha (TRA@), but also genes with unknown function. The network in figure 4 summarizes and visualizes the effect of diclofenac on inflammation: inhibition of prostaglandin synthesis and changes in associated oxylipids, together with inhibition of TNF-alpha and closely related genes and proteins like caspase 8.

## Discussion

Obesity is often associated with elevated indicators of a chronic inflammatory state. However, many common inflammation markers report on different aspects of this condition, highlighting that inflammation is complex and multifactorial. In this study of moderately overweight subjects, we investigated the impact of a 9-day standard dose of the NSAID diclofenac on a broad array of transcriptomic, proteomic and metabolomic markers, including many common markers of inflammatory status. A recognized mechanism of NSAID antipyretic, analgesic and anti-inflammatory efficacy is through the blockade of prostaglandin synthesis by inhibition of cycooxygenase enzymes. Consistent with this mechanism, a reduction in plasma PGE_2 _levels was observed in the diclofenac treatment group. Additionally, diclofenac intervention resulted in reduced levels of inflammation marker TNF-alpha.

Established inflammatory markers are mostly associated with obesity-related diseases like cardiovascular disease and type-2 diabetes [9,13,16]. The subjects in the current study were overweight and relatively healthy, i.e. before manifestation of obesity-related diseases. The lack of response of most of these inflammatory markers other than PGE_2 _to modulation of inflammation by diclofenac and the interference of acute phase response markers in the placebo group clearly demonstrates the need for more detailed and accurate descriptors of low-grade inflammatory status and its modulation, specifically associated with mild obesity. This study identified a panel of genes, proteins and metabolites that describe a diclofenac-response network in overweight subjects. This panel contains potentially more stable and sensitive markers of modulation of low-grade inflammation in overweight subjects, as compared to the established markers that often also respond to an acute inflammatory challenge. As indicated in figure 4, many of these novel markers appear functionally related to the accepted mode of action of diclofenac. Inhibition of prostaglandin synthesis results in higher levels of arachidonic acid and its oxylipid metabolites 5,6-DHET and 20-HETE. The intermediate metabolite 5(6)-epoxy eicosatrienoic acid (5,(6)-EET) is a potent modulator of ion conductance in various tissues, and its biological effects are often COX-dependent [28-30]. While no change in PBMC COX-2 expression was detected, annexin A1 (ANXA1) expression was induced in response to diclofenac, increasing 64 ± 41% (mean ± stdev) in this cell type. This anti-inflammatory protein can inhibit enzymes phospholipase A2 and COX-2, thereby inhibiting prostaglandin synthesis [31]. TNF alpha can also affect prostaglandin synthesis by inducing expression of prostaglandin synthase [32]. Network analysis identified diclofenac responsive genes T cell receptor alpha, caspase 8 and AKAP13 as markers functionally related to TNF alpha, as shown in figure 4. It is known that NSAID, including diclofenac, can suppress TNF alpha induced NFkB activation [33] and that NFkB can regulate prostaglandin synthesis through COX-2 [34]. The observed changes in PGE_2_, TNF alpha and annexin A2 corroborate anti-inflammatory effects of the diclofenac treatment in the overweight subjects. Furthermore, more detailed biological network analysis allowed us to identify and visualize functionally related diclofenac responsive genes, proteins and metabolites. The markers in the network may each be sensitive markers of inflammatory modulation. However, it is important to note that a considerable number of diclofenac responsive genes are genes not previously associated with inflammation and genes with unknown function. Although these genes can therefore not be included in the biological network analysis, each of these could be potential interesting links for novel markers of inflammation associated with obesity.

Interestingly, the majority of the metabolites measured did not show a differential response between the diclofenac and the placebo group, with the exception of the metabolites of the oxylipids platform. However, in addition to measurements in fasting condition, the response of metabolites was also studied during an oral glucose tolerance test, performed at the beginning and the end of the study, based on the idea that by challenging a system you can test its resilience. In the face of this metabolic challenge test, subtle diclofenac-responsive changes were detected, e.g. reduced plasma levels of uric acid and changes in levels of metabolites from the insulin regulated glutathione synthesis pathway. These findings are described in detail elsewhere [21].

Diclofenac intervention did not affect levels of the known inflammation markers CRP, sialic acid, VCAM or ICAM. Influences on plasma IL-6 could not be determined since levels were below the detection limit in the multiplex proteome analysis. In the placebo group, large within-subject variation of CRP was observed (Figure 2). This probably reflects the function of CRP as an acute phase protein and data suggested that a subset of subjects in the placebo group had unrecognized acute phase response underway at study day 0. In addition to the fluctuation over time, it should be noted that CRP levels also showed large between-subject variation (table 1, figure 2). It was evaluated whether this acute response of CRP was reflected in the transcriptome, proteome and metabolome data sets by performing correlation analysis on CRP response and the gene, protein and oxylipid responses in all subjects. The network in additional file 5 illustrates biological connections between CRP and the most highly correlated genes and proteins (MetaCore™). The identified network of genes/proteins contains multiple nodes with roles in the acute phase response: complement 3 (C3), ferritin, PAI-1 [35]. Furthermore, the transcription factor STAT3, which can activate CRP transcription [12], has a central position in the network. The markers in this inflammation network may be more relevant as markers of fluctuations in acute inflammation than as markers of inflammatory modulation by pharmaceutical or nutritional intervention.

The panel of genes, proteins and metabolites modulated by diclofenac in this study appear to provide alternative measures of inflammatory status when associated with mild chronic conditions as observed in obesity. By using these markers to assess subject responsiveness prior to subject selection, the resulting reduction in variance is expected to increase the statistical power of studies designed to assess inflammatory intervention strategies in such chronic mild conditions. This increased power might then result in detection of changes in conventional inflammatory markers that were not detected in this study. It should be noted that larger subject groups could also contribute to increased power to detect changes in inflammatory markers. Furthermore, it should be noted that additional studies are needed to determine whether the markers identified here are specific for modulation of inflammation through inhibition of prostaglandin synthesis or if they are more general markers of inflammatory modulation. Ultimately, this study shows how the analysis and integration of a wide-range of parameters can lead to the selection of both known and new markers that respond to inflammatory modulation.

## Conclusion

We report an array of new potential markers of inflammatory responsiveness to NSAID therapy in overweight subjects that include PBMC expression of ANXA1 and caspase 8, as well as plasma concentrations of 5,6-DHET. These findings constitute an advance in our ability to understand and quantify the status and modulation of inflammation in humans.

## Competing interests

The authors declare that they have no competing interests.

## Authors' contributions

MvE was responsible for generation and analysis of the microarray data, performed data integration and interpretation and drafted the manuscript; EV and SW were responsible for metabolomics measurements and metabolomics data analysis; TP and JN were responsible for oxylipids measurements; CR and AS performed the multivariate analysis. SW, CR, TP, JN, NP, AS, HH and BvO helped to draft the manuscript. TvV, NP, HH, JvdG and BvO designed the study. All authors read and approved the manuscript.

## Pre-publication history

The pre-publication history for this paper can be accessed here:

http://www.biomedcentral.com/1755-8794/3/5/prepub

## Supplementary Material

Additional file 1 **Overview of plasma proteins**. List of inflammation related plasma proteins measured in multiplex analysis. Click here for file

Additional file 2 **Changes in plasma oxylipids**. Mean (± stdev) and median % change of plasma oxylipids selected by PLS-DA Click here for file

Additional file 3 **Changes in plasma proteins**. Mean (± stdev) and median % change of plasma proteins selected in PLS-DA Click here for file

Additional file 4 **Enriched biological processes in selected set of genes**. Enriched biological processes (based on Gene Ontology) in selected set of genes. Selected set of genes was analyzed in GenMAPP v2.0. Biological processes are ordered by Z-score. Click here for file

Additional file 5 **Network showing biological connections between genes and protein selected on highest correlation of expression change to CRP change**. Network showing biological connections between genes and protein selected on highest correlation of expression change to CRP change (day 9 vs. day 0, n = 18). Network was generated using curated interactions in MetaCore v4.7 (GeneGo Inc., St. Joseph, MI, USA). Click here for file
