# Supplementary material for: Insight in modulation of inflammation in response to diclofenac intervention: a human intervention study
Source: BMC Med Genomics. 2010 Feb 23;3:5. doi: 10.1186/1755-8794-3-5 (PMC2837611; doi:10.1186/1755-8794-3-5)
Supplement: Additional file 1 — Overview of plasma proteins. List of inflammation related plasma proteins measured in multiplex analysis. [file 1755-8794-3-5-S1.PDF]

Additional file 1.
Proteins in multiplex analysis

1 Adiponectin
2 Alpha-1 Antitrypsin
3 Alpha-2 Macroglobulin
4 Alpha-Fetoprotein
5 Apolipoprotein A1
6 Apolipoprotein CIII
7 Apolipoprotein H
8 Beta-2 Microglobulin
9 Brain-Derived Neurotrophic Factor
10 C Reactive Protein
11 Calcitonin (*)
12 Cancer Antigen 125 (*)
13 Cancer Antigen 19-9
14 Carcinoembryonic Antigen
15 Complement 3
16 Creatine Kinase-MB (*)
17 EGF (*)
18 ENA-78
19 Endothelin-1
20 Eotaxin
21 Erythropoietin (*)
22 Factor VII
23 Fatty Acid Binding Protein (*)
24 Ferritin
25 FGF basic (*)
26 Fibrinogen
27 Glutathione S-Transferase (*)
28 GM-CSF
29 Growth Hormone (*)
30 ICAM-1
31 IgA
32 IgE
33 IgM
34 IL-10
35 IL-12p40
36 IL-12p70
37 IL-13
38 IL-15
39 IL-16
40 IL-18
41 IL-1alpha
42 IL-1beta (*)
43 IL-2 (*)
44 IL-3
45 IL-4
46 IL-5
47 IL-6 (*)
48 IL-7
49 IL-8
50 Insulin
51 Leptin
52 Lipoprotein (a)
53 Lymphotactin
54 MCP-1
55 MDC
56 MIP-1alpha
57 MIP-1beta
58 MMP-2
59 MMP-3 (*)
60 MMP-9
61 Myoglobin
62 PAI-1
63 Prostate Specific Antigen, Free
64 Prostatic Acid Phosphatase
65 RANTES
66 Serum Amyloid P
67 SGOT
68 Stem Cell Factor
69 Thrombopoietin
70 Thyroid Stimulating Hormone
71 Thyroxine Binding Globulin
72 TIMP-1
73 Tissue Factor
74 TNF RII
75 TNF-alpha
76 TNF-beta
77 VCAM-1
78 VEGF
79 von Willebrand Factor

(*) not included in data analysis due to measurements below detection limit
