# Supplementary material for: Insight in modulation of inflammation in response to diclofenac intervention: a human intervention study
Source: BMC Med Genomics. 2010 Feb 23;3:5. doi: 10.1186/1755-8794-3-5 (PMC2837611; doi:10.1186/1755-8794-3-5)
Supplement: Additional file 2 — Changes in plasma oxylipids. Mean (± stdev) and median % change of plasma oxylipids selected by PLS-DA [file 1755-8794-3-5-S2.PDF]

Additional file 2. Mean (± stdev) and median of % change for plasma oxylipids selected by PLS-DA

Rank PLS	Name	placebo Day 0	placebo Day 9	median % change	diclofenac Day 0	diclofenac Day 9	median % change
1	_5_6_DHET (*)	0.54 ± 0.25	0.51 ± 0.14		0.48 ± 0.16	0.66 ± 0.26	50
2	_20_HETE (*)	0.78 ± 0.30	0.74 ± 0.32		0.62 ± 0.18	1.00 ± 0.47	57
3	_12_HETE (*)	5.87 ± 3.09	9.59 ± 6.62	43	6.43 ± 4.21	5.73 ± 4.99	
4	_9_10_DHOME (*)	4.26 ± 1.38	4.91 ± 2.10		7.28 ± 7.04	3.95 ± 1.45	-38
5	_12_13_DHOME	6.71 ± 1.89	8.53 ± 3.46		7.45 ± 3.32	6.61 ± 1.63	
6	_6_keto_PGF1a	1.70 ± 0.22	1.59 ± 0.15		1.68 ± 0.13	1.85 ± 0.57	
7	_9_HETE	0.78 ± 0.45	0.80 ± 0.53		0.51 ± 0.19	0.94 ± 0.68	79
8	_12_13__EpOME	2.47 ± 1.12	3.11 ± 2.53		2.66 ± 1.36	2.64 ± 1.75	
9	_11_12__EET	0.24 ± 0.09	0.23 ± 0.06		0.18 ± 0.07	0.42 ± 0.51	
10	_9_oxo_ODE	2.96 ± 2.79	2.53 ± 0.97	37	1.65 ± 1.06	3.71 ± 3.87	30
11	_14_15__EET	0.27 ± 0.12	0.26 ± 0.07		0.27 ± 0.10	0.38 ± 0.20	
12	_8_HETE	0.77 ± 0.36	0.90 ± 0.48		0.64 ± 0.28	1.05 ± 0.86	64
13	_9_10__EpOME	2.22 ± 1.22	1.90 ± 0.54		2.00 ± 1.27	3.02 ± 2.71	
14	_11_12_DHET	1.18 ± 0.39	1.30 ± 0.20		1.18 ± 0.41	1.13 ± 0.22	
15	_15_HETE	3.67 ± 1.14	3.93 ± 1.08		3.22 ± 0.88	4.26 ± 2.18	
16	_9_10_13_TriHOME	1.16 ± 0.59	1.17 ± 0.45		0.97 ± 0.39	1.90 ± 2.10	
17	_5_oxo_ETE	0.50 ± 0.30	0.45 ± 0.23		0.36 ± 0.20	0.56 ± 0.39	
18	_14_15_DHET	1.14 ± 0.26	1.27 ± 0.20		1.15 ± 0.31	1.12 ± 0.19	
19	_13_HODE	36.90 ± 16.04	42.96 ± 11.66	25	36.70 ± 14.33	45.51 ± 22.00	

Median % change: For each subject, % change was calculated from fold change (day 9/day 0). Median value of % change (day 9 vs day 0) in the subjects in the placebo group or in subjects in the diclofenac group is listed for oxylipids if % change was >20% in 6 or more subjects in the group or if % change was <-20% in 6 or more subjects in the group. Median values are reported as these are more robust to variation in response between subjects.

(*): 2-way ANOVA p-value for time x treatment interaction <0.1
