# Supplementary material for: Insight in modulation of inflammation in response to diclofenac intervention: a human intervention study
Source: BMC Med Genomics. 2010 Feb 23;3:5. doi: 10.1186/1755-8794-3-5 (PMC2837611; doi:10.1186/1755-8794-3-5)
Supplement: Additional file 3 — Changes in plasma proteins. Mean (± stdev) and median % change of plasma proteins selected in PLS-DA [file 1755-8794-3-5-S3.PDF]

Additional file 3. Mean (± stdev) and median of % change for plasma proteins selected in PLS-DA

Rank PLS	Name		placebo day0	placebo day9	median % change	diclofenac day0	diclofenac day9	median % change
1	Thyroxine Binding Globulin (*)	ug/mL	45.8 ± 9.1	45.7 ± 8.3		49.0 ± 8.0	45.3 ± 7.5	
2	Ferritin (*)	ng/mL	153 ± 87	128 ± 79		225 ± 141	268 ± 198	
3	MIP-1alpha	pg/mL	12.2 ± 2.2	10.5 ± 4.2	-33	10.8 ± 2.0[a]	11.5 ± 8.0[a]	
4	Prostate Specific Antigen, Free	ng/mL	0.35 ± 0.19	0.41 ± 0.23		0.30 ± 0.08	0.30 ± 0.08	
5	C Reactive Protein (*)	ug/mL	4.9 ± 4.3	2.3 ± 2.5	-68	1.7 ± 1.1	2.2 ± 2.0	
6	Alpha-2 Macroglobulin (*)	mg/mL	0.13 ± 0.02	0.15 ± 0.03	23	0.15 ± 0.04	0.12 ± 0.03	
7	Cancer Antigen 19-9	U/mL	1.26 ± 0.79[b]	1.23 ± 0.69[b]		0.96 ± 0.51[c]	0.95 ± 0.45[c]	
8	Serum Amyloid P	ug/mL	28.7 ± 7.4	26.3 ± 7.0		28.3 ± 5.1	28.0 ± 6.4	
9	PAI-1 (*)	ng/mL	81.4 ± 26.5	63.8 ± 18.5	-31	62.7 ± 17.5	62.0 ± 29.9	
10	TIMP-1 (*)	ng/mL	68.5 ± 11.3	72.8 ± 7.4		73.0 ± 5.2	69.2 ± 8.5	
11	MIP-1beta	pg/mL	149 ± 33	133 ± 26		133 ± 33	131 ± 30	
12	ENA-78 (*)	ng/mL	0.12 ± 0.10	0.23 ± 0.23	80	0.16 ± 0.12[a]	0.13 ± 0.07[a]	
13	SGOT (*)	ug/mL	15.4 ± 2.3	15.8 ± 2.1		15.1 ± 2.4	17.2 ± 2.1	
14	TNF-beta	pg/mL	56.3 ± 17.2	53.8 ± 19.6		47.6 ± 12.9	57.7 ± 26.0	
15	GM-CSF	pg/mL	63.9 ± 15.0	64.1 ± 28.1		61.0 ± 18.2	70.8 ± 21.7	
16	IL-15	ng/mL	1.9 ± 0.6	1.8 ± 0.5		2.0 ± 0.5	1.6 ± 0.5	
17	Fibrinogen	mg/mL	3.4 ± 0.5	3.0 ± 0.5		3.5 ± 0.6	2.9 ± 0.7	
18	Factor VII (*)	ng/mL	273 ± 50	266 ± 47		305 ± 48	269 ± 40	
19	MDC	pg/mL	332 ± 116	303 ± 130		393 ± 110	328 ± 101	
20	Beta-2 Microglobulin	ug/mL	1.6 ± 0.3	1.6 ± 0.3		1.8 ± 0.3	1.7 ± 0.3	
21	RANTES	ng/mL	1.8 ± 0.7	4.2 ± 3.8	93	2.4 ± 2.0	2.5 ±1.6	78
22	IgA	mg/mL	2.0 ± 0.8	1.9 ± 0.6		1.7 ± 0.9	1.5 ± 0.7	
23	TNF RII	ng/mL	4.1 ± 0.7	3.7 ± 0.4		3.7 ±0.6	3.7 ± 0.7	
24	Thrombopoietin	ng/mL	4.0 ± 2.6	3.5 ± 2.3		2.6 ± 1.2	2.6 ± 1.2	
25	Carcinoembryonic Antigen	ng/mL	0.87 ± 0.74	0.91 ± 0.61		0.57 ± 0.43	0.52 ± 0.32	
26	IgM	mg/mL	0.67 ± 0.27	0.66 ± 0.31		0.83 ± 0.33	0.73 ± 0.30	
27	Alpha-Fetoprotein (*)	ng/mL	1.6 ± 1.1	1.7 ± 0.9		1.5 ± 0.4	1.3 ± 0.5	
28	IL-3	ng/mL	0.71 ± 0.30	0.71 ± 0.37		0.67 ± 0.18	0.80 ± 0.26	
29	TNF-alpha (*)	pg/mL	2.0 ± 2.8	2.9 ± 3.0	61	2.1 ± 0.6	1.5 ± 1.1	-171
30	IL-18	pg/mL	251 ± 72	228 ± 51		261 ± 85	247 ± 79	
31	Tissue Factor	ng/mL	0.34 ± 0.15	0.32 ± 0.16		0.35 ± 0.10	0.33 ± 0.14	
32	IL-5	pg/mL	26.3 ± 5.5	26.0 ± 14.7		20.8 ± 6.1	24.5 ± 11.4	
33	VEGF	pg/mL	167 ± 69	154 ± 56		165 ± 93	147 ± 82	
34	Brain-Derived Neurotrophic Factor	ng/mL	0.37 ± 0.21	0.69 ± 0.40	91	0.36 ± 0.28	0.45 ± 0.31	
35	IL-8	pg/mL	20.2 ± 7.0	17.2 ± 3.5		16.0 ± 4.0	13.9 ± 4.7	
36	Prostatic Acid	ng/mL	0.13 ± 0.02	0.13 ± 0.03		0.15 ± 0.03	0.13 ± 0.03	

	Phosphatase							
37	MCP-1	pg/mL	161 ± 55	145 ± 27		176 ± 24	164 ± 39	
38	Apolipoprotein A1	mg/mL	0.25 ± 0.06	0.24 ± 0.05		0.25 ± 0.04	0.22 ± 0.03	
39	Thyroid Stimulating Hormone	uIU/mL	1.1 ± 0.6	1.2 ± 0.4		1.3 ± 0.3	1.2 ± 0.3	
40	IL-10	pg/mL	19.4 ± 4.0	16.6 ± 4.7	-30	20.1 ± 2.9	18.4 ± 5.9	
41	Lipoprotein (a)	ug/mL	66.5 ± 63.7	73.7 ± 86.8		114.2 ± 163.6	112.0 ± 140.4	
42	IL-12p40	ng/mL	1.1 ± 0.4	1.2 ± 0.6		0.9 ± 0.2	1.1 ± 0.4	
43	Apolipoprotein H	ug/mL	187 ± 47	181 ± 41		173 ± 59	158 ± 52	
44	IL-1alpha	ng/mL	0.44 ± 0.11	0.46 ± 0.15		0.42 ± 0.07	0.44 ± 0.10	
45	IL-4	pg/mL	55.6 ± 12.9	52.1 ± 9.9		54.1 ± 13.2	44.5 ± 11.4	
46	Apolipoprotein CIII	ug/mL	48.4 ± 19.9	42.6 ± 11.6		54.9 ± 12.3	42.7 ± 10.7	-29

Median % change: For each subject, % change was calculated from fold change (day 9/day 0). Median value of % change (day 9 vs day 0) in the subjects in the placebo group or in subjects in the diclofenac group is listed for proteins if % change was >20% in 6 or more subjects in the group or if % change was <-20% in 6 or more subjects in the group. Median values are reported as these are more robust to variation in response between subjects.

For example: Changes in TNF-alpha for subjects in the diclofenac group were: -306%, -238.5%, -238.5%, -238.5%, -171%, -25%, 0%, +7.7%, +438.5% (as shown in figure 3). Median value for change in TNF-alpha: -171%.

(*): 2-way ANOVA p-value for time x treatment interaction <0.1

[a] : Average and stdev calculated for 8 subjects, proteins was not detected in 1 subject

[b] : Average and stdev calculated for 9 subjects, proteins was not detected in 1 subject

[c] : Average and stdev calculated for 7 subjects, proteins was not detected in 2 subjects
