# Supplementary material for: Insight in modulation of inflammation in response to diclofenac intervention: a human intervention study
Source: BMC Med Genomics. 2010 Feb 23;3:5. doi: 10.1186/1755-8794-3-5 (PMC2837611; doi:10.1186/1755-8794-3-5)
Supplement: Additional file 4 — Enriched biological processes in selected set of genes. Enriched biological processes (based on Gene Ontology) in selected set of genes. Selected set of genes was analyzed in GenMAPP v2.0. Biological processes are ordered by Z-score. [file 1755-8794-3-5-S4.PDF]

Additional file 4. Enriched biological processes (based on Gene Ontology) in selected set of genes

Biological processes with size 6 to 1000 genes were included

GO Biological Process	Number of genes measured	Number of genes changed	Percent Changed	Z Score	PermuteP
response to biotic stimulus	810	164	20.25	7.43	0.000
immune response	688	142	20.64	7.14	0.000
defense response	772	155	20.08	7.09	0.000
response to pest\, pathogen or parasite	472	95	20.13	5.52	0.000
response to stress	899	157	17.46	5.20	0.000
response to other organism	499	97	19.44	5.20	0.000
protein kinase cascade	259	57	22.01	4.99	0.000
inflammatory response	191	42	21.99	4.27	0.000
I-kappaB kinase/NF-kappaB cascade	107	27	25.23	4.22	0.000
regulation of I-kappaB kinase/NF-kappaB cascade	81	22	27.16	4.20	0.000
positive regulation of cellular process	445	81	18.20	4.08	0.000
positive regulation of signal transduction	89	23	25.84	4.02	0.000
positive regulation of biological process	517	91	17.60	3.98	0.000
nucleotide-sugar metabolism	16	7	43.75	3.91	0.002
cell death	463	82	17.71	3.83	0.000
response to wounding	344	64	18.60	3.81	0.001
programmed cell death	438	78	17.81	3.79	0.000
positive regulation of I-kappaB kinase/NF-kappaB cascade	77	20	25.97	3.78	0.000
death	467	82	17.56	3.75	0.000
autophagy	17	7	41.18	3.70	0.002
apoptosis	435	76	17.47	3.56	0.000
establishment of protein localization	529	89	16.82	3.47	0.001
protein localization	543	91	16.76	3.47	0.000
antimicrobial humoral response (sensu Vertebrata)	82	20	24.39	3.46	0.001
protein transport	509	86	16.90	3.45	0.001
modification-dependent macromolecule catabolism	117	26	22.22	3.41	0.001
modification-dependent protein catabolism	117	26	22.22	3.41	0.001
ubiquitin-dependent protein catabolism	117	26	22.22	3.41	0.001
positive regulation of programmed cell death	136	29	21.32	3.35	0.002
antimicrobial humoral response	84	20	23.81	3.33	0.001
pyridine nucleotide metabolism	19	7	36.84	3.33	0.002
response to external stimulus	446	76	17.04	3.32	0.001
transition metal ion transport	37	11	29.73	3.32	0.004
regulation of programmed cell death	264	49	18.56	3.30	0.004
humoral immune response	151	31	20.53	3.24	0.000
positive regulation of physiological process	375	65	17.33	3.21	0.005
NLS-bearing substrate import into nucleus	12	5	41.67	3.16	0.006
energy derivation by oxidation of organic compounds	141	29	20.57	3.14	0.003
protein amino acid acylation	16	6	37.50	3.14	0.009
positive regulation of apoptosis	135	28	20.74	3.13	0.003
striated muscle development	34	10	29.41	3.12	0.007
cellular macromolecule catabolism	271	49	18.08	3.10	0.004
hexose metabolism	124	26	20.97	3.08	0.003
induction of apoptosis by extracellular signals	30	9	30.00	3.03	0.009
positive regulation of cellular physiological process	363	62	17.08	3.01	0.004
transition metal ion homeostasis	21	7	33.33	3.01	0.010
macromolecule biosynthesis	582	93	15.98	3.01	0.001
regulation of apoptosis	261	47	18.01	3.01	0.004
pentose-phosphate shunt	9	4	44.44	2.99	0.015
NADPH regeneration	9	4	44.44	2.99	0.015
ER-associated protein catabolism	9	4	44.44	2.99	0.029
misfolded or incompletely synthesized protein catabolism	9	4	44.44	2.99	0.029
ubiquitin cycle	513	83	16.18	2.96	0.002
monosaccharide metabolism	127	26	20.47	2.94	0.004
protein targeting	140	28	20.00	2.92	0.001
virus-host interaction	6	3	50.00	2.86	0.028
pyrimidine nucleoside metabolism	6	3	50.00	2.86	0.031
monosaccharide biosynthesis	22	7	31.82	2.86	0.017

GO Biological Process	Number of genes measured	Number of genes changed	Percent Changed	Z Score	PermuteP
alcohol biosynthesis	22	7	31.82	2.86	0.017
hexose biosynthesis	22	7	31.82	2.86	0.017
induction of apoptosis	125	25	20.00	2.76	0.005
induction of programmed cell death	125	25	20.00	2.76	0.005
copper ion transport	10	4	40.00	2.72	0.022
NADP metabolism	10	4	40.00	2.72	0.023
oxidoreduction coenzyme metabolism	23	7	30.43	2.72	0.012
main pathways of carbohydrate metabolism	96	20	20.83	2.67	0.015
humoral defense mechanism (sensu Vertebrata)	109	22	20.18	2.63	0.012
iron ion transport	19	6	31.58	2.62	0.012
proteolysis during cellular protein catabolism	148	28	18.92	2.60	0.012
protein biosynthesis	518	81	15.64	2.58	0.006
intracellular protein transport	339	56	16.52	2.58	0.004
JAK-STAT cascade	29	8	27.59	2.58	0.011
cellular protein catabolism	149	28	18.79	2.56	0.012
glucose metabolism	86	18	20.93	2.55	0.020
nicotinamide metabolism	15	5	33.33	2.54	0.027
apoptotic mitochondrial changes	7	3	42.86	2.51	0.034
regulation of GTPase activity	35	9	25.71	2.50	0.019
cellular defense response	88	18	20.45	2.44	0.023
cellular carbohydrate metabolism	283	47	16.61	2.40	0.020
immune cell activation	89	18	20.22	2.39	0.020
cellular catabolism	412	65	15.78	2.38	0.020
macromolecule catabolism	305	50	16.39	2.38	0.026
regulation of cell differentiation	42	10	23.81	2.35	0.023
cell activation	90	18	20.00	2.34	0.022
regulation of signal transduction	196	34	17.35	2.31	0.028
muscle cell differentiation	12	4	33.33	2.27	0.043
lymphocyte activation	73	15	20.55	2.25	0.030
anti-apoptosis	86	17	19.77	2.22	0.034
peroxisome organization and biogenesis	17	5	29.41	2.21	0.042
negative regulation of programmed cell death	99	19	19.19	2.20	0.036
phagocytosis	22	6	27.27	2.20	0.026
protein import into nucleus	62	13	20.97	2.17	0.029
nuclear import	62	13	20.97	2.17	0.029
alcohol metabolism	229	38	16.59	2.15	0.043
Notch signaling pathway	28	7	25.00	2.12	0.040
regulation of enzyme activity	211	35	16.59	2.06	0.040
glucose catabolism	58	12	20.69	2.04	0.048
regulation of development	59	12	20.34	1.97	0.049
