# Supplementary material for: Insight in modulation of inflammation in response to diclofenac intervention: a human intervention study
Source: BMC Med Genomics. 2010 Feb 23;3:5. doi: 10.1186/1755-8794-3-5 (PMC2837611; doi:10.1186/1755-8794-3-5)

Additional file 5. Network showing biological connections between genes and protein selected on highest correlation of expression change to CRP change (day 9 vs day 0, n=18)

16 genes (correlation > |0.8|) and 1 protein (correlation to CRP change +0.75) were submitted to network analysis (shown as encircled nodes). Numbers indicate Pearson correlation coefficient to CRP change, (P) indicates correlation coefficient for protein data, all other correlation coefficients are for PBMC gene expression data. CR1: complement receptor 1; C3: complement 3; SHPS-1: Tyrosine-protein phosphatase non-receptor type substrate 1 precursor; SHP1: Tyrosine-protein phosphatase non-receptor type 6; GRB2: Growth factor receptor-bound protein 2; Rab5ip: Uncharacterized protein C20orf24; ESR1: estrogen receptor 1; HSF1: heat shock factor protein 1; STAT3: signal transducer and activator of transcription 3; C/EBPdelta: CCAAT/enhancer binding protein delta; ALDH2: aldehyde dehydrogenase 2; C/EPBalpha: CCAAT/enhancer binding protein alpha; CD13: aminopeptidase N; CBP: CREB binding protein; PAI1: plasminogen activator inhibitor 1 precursor; CRP: C reactive protein; Bcl-6: B-cell lymphoma 6 protein; EGFR: epidermal growth factor receptor.

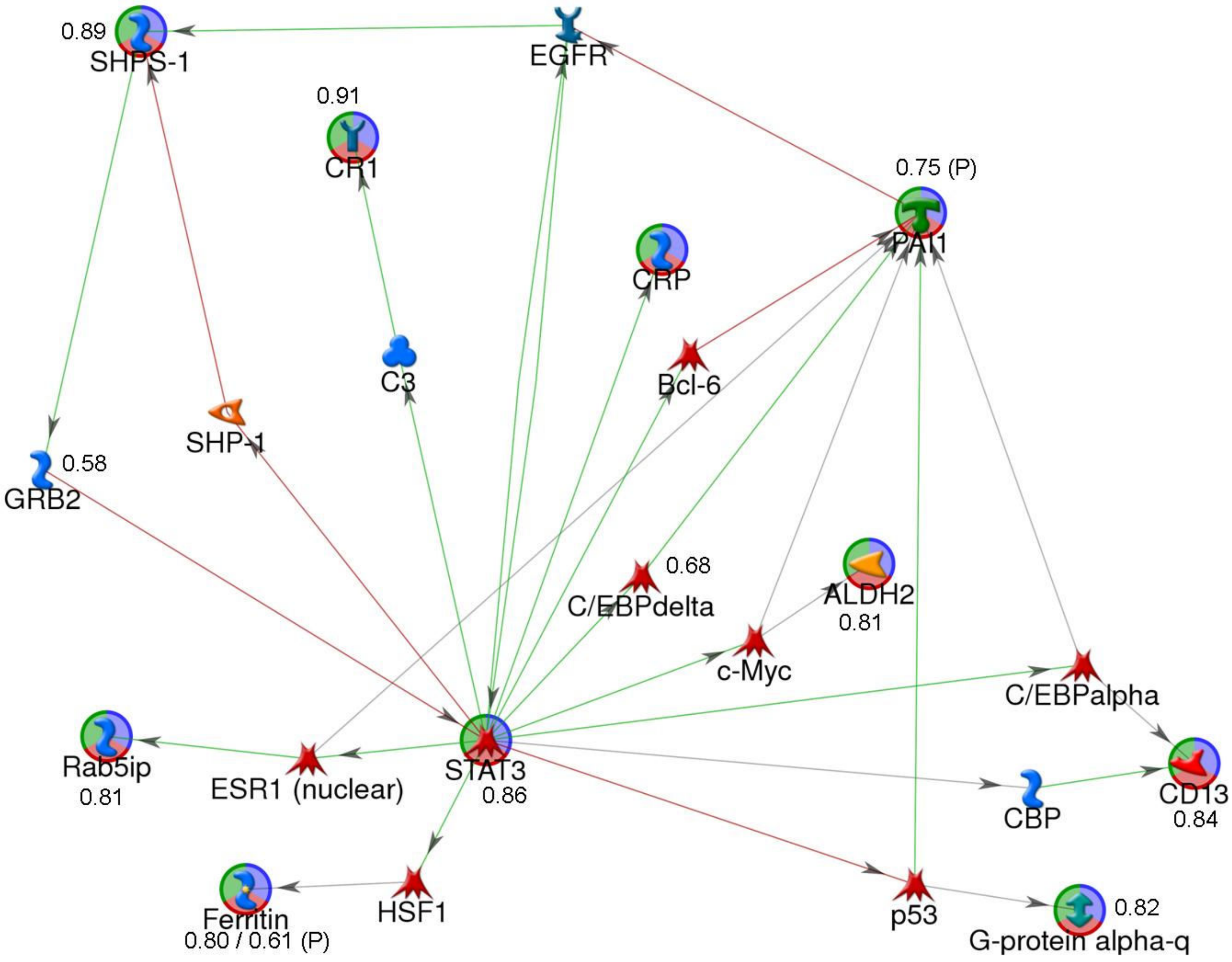

Supplement: Additional file 5 — Network showing biological connections between genes and protein selected on highest correlation of expression change to CRP change. Network showing biological connections between genes and protein selected on highest correlation of expression change to CRP change (day 9 vs. day 0, n = 18). Network was generated using curated interactions in MetaCore v4.7 (GeneGo Inc., St. Joseph, MI, USA). [file 1755-8794-3-5-S5.PDF]
